# Thinking in Terms of Change over Time: Opportunities and Challenges of Using System Dynamics Models

**DOI:** 10.1007/s10956-023-10047-y

**Published:** 2023-06-13

**Authors:** Emil Eidin, Tom Bielik, Israel Touitou, Jonathan Bowers, Cynthia McIntyre, Dan Damelin, Joeseph Krajcik

**Affiliations:** 1 CREATE for STEM, Michigan State University, East Lansing, MI USA; 2 The Concord Consortium, Concord, MA USA; 3 Freie Universität-Berlin, Berlin, Germany

**Keywords:** Modeling, Systems thinking, System dynamics, Chemical kinetics

## Abstract

Understanding the world around us is a growing necessity for the whole public, as citizens are required to make informed decisions in their everyday lives about complex issues. Systems thinking (ST) is a promising approach for developing solutions to various problems that society faces and has been acknowledged as a crosscutting concept that should be integrated across educational science disciplines. However, studies show that engaging students in ST is challenging, especially concerning aspects like change over time and feedback. Using computational system models and a system dynamics approach can support students in overcoming these challenges when making sense of complex phenomena. In this paper, we describe an empirical study that examines how 10th grade students engage in aspects of ST through computational system modeling as part of a Next Generation Science Standards-aligned project-based learning unit on chemical kinetics. We show students’ increased capacity to explain the underlying mechanism of the phenomenon in terms of change over time that goes beyond linear causal relationships. However, student models and their accompanying explanations were limited in scope as students did not address feedback mechanisms as part of their modeling and explanations. In addition, we describe specific challenges students encountered when evaluating and revising models. In particular, we show epistemological barriers to fruitful use of real-world data for model revision. Our findings provide insights into the opportunities of a system dynamics approach and the challenges that remain in supporting students to make sense of complex phenomena and nonlinear mechanisms.

## Introduction

Systems thinking, an approach used in many fields and across disciplines, supports understanding complex phenomena and solving challenging problems. Science educators have been advocating in recent years for the integration of systems thinking in science education as part of an endeavor to prepare scientifically literate citizens who are equipped with thinking skills that would support them in making sense of the complex phenomena they experience in everyday life (Arndt, 2006; Assaraf et al., 2005; National Research Council [NRC], 2012). Modeling has been advocated as a promising practice that supports students in applying ST to make sense of a phenomenon (Arndt, 2006; Eilam, 2012; Yoon & Hmelo-Silver, 2017), and previous work has demonstrated how modeling practices are aligned with aspects of systems thinking (ST) (Shin et al., 2022). In particular, computational models provide a promising avenue that supports students’ ST to make sense of various phenomena, given the affordances that allow the computation of a web of interactions that would be very different to predict and interpret otherwise (Mandinach, 1989; Richmond, 1993). The authors’ work revealed opportunities and challenges about aspects of ST students encountered during the modeling process as they use computational system modeling to make sense of a phenomenon. Building off that work, we broaden several of the ST aspects that are aligned with and informed by the modeling process and deepen the examination of opportunities and challenges of using computational system models.

Modeling is a critical practice used by scientists in their everyday work to make sense of the world and produce new knowledge. Indeed, modeling is one of the key science and engineering practices promoted in *A Framework for K-12 Science Education* (NRC, 2012) and the Next Generation Science Standards (NGSS Lead States, 2013) for K-12 science education. It is, therefore, essential that students are equipped with the epistemic foundations of the modeling process and are able to understand and evaluate scientific models (Acher et al., 2007; Louca & Zacharia, 2012) as preparation for active participation in public discourse (Ke et al., 2021). Lack of explicit preparation with the aforementioned practices as well as nature of science and scientific habits of mind (NRC, 2012; Osborne, 2014) may lead to misunderstanding, misinterpretation, and even denial of scientific research, as in the case of climate change and COVID-19 (Sinatra & Hofer, 2021).

Scholars distinguish between students using pre-existing models and students constructing their own models. The latter positions students as agents of knowledge who explain phenomena and solve problems (Lehrer et al., 2006; Schwarz et al., 2009). The modeling process is generally broken down into four practices: constructing, evaluating, revising, and using models (Schwarz et al., 2009). The advantages of students constructing models are broadly documented, including exposing learners to a realistic epistemological view of scientific knowledge and the nature and purpose of models as both sources of evidence and exploratory tools (Harrison & Treagust, 2000; Schwarz et al., 2017), supporting students in making sense of a phenomenon (Acher et al., 2007; Windschitl et al., 2008), and giving students ownership of the modeling process and what they are figuring out (Stroupe, 2014).

Rapid technological improvements in recent years have enabled the introduction of computational modeling tools, offering students a suite of full-fledged tools to engage in modeling practices in which students can construct, evaluate, revise, and use their models (Bielik et al., 2019; Clark & Ernst, 2008). Yet, despite the promising results of integrating computational models, applying ST in model construction still poses a challenge for students (Chi et al., 2012; Jacobson & Wilensky, 2006; Tripto et al., 2018). Moreover, there is no description of how those challenges are aligned, if at all, with the modeling process and specific modeling practices. In this paper, we seek to describe the opportunities and challenges students encounter in applying ST during a model-building experience to make sense of scientific phenomena.

## Theoretical Background

### Systems Thinking

Systems thinking is a cognitive skill essential to supporting students in their efforts to make sense of complex phenomena (Assaraf & Orion, 2005; Kali et al., 2003; Mathews et al., 2008) and has guided curriculum design policy (KMK, 2005; NGSS Lead States, 2013). For example, systems and system models represent one of the crosscutting concepts in *A Framework for K-12 Science Education* (NRC, 2012), which acknowledges systems as both fundamental to scientific thinking and a critical component of science education. There is a consensus about prominent aspects of ST and their practical manifestations (Assaraf et al., 2013; Hmelo-Silver et al., 2007; Sweeney & Sterman, 2000). A recent literature review (Shin et al., 2022) identifies five ST aspects that are common to a large body of work on ST.

1. *Defining a system* (boundaries and structure) requires identifying relevant components that make up a system, including specifying its inputs and outputs (Arnold & Wade, 2017; Assaraf & Orion, 2005; Stave & Hopper, 2007).
2. *Framing problems or phenomena in terms of behavior over time* involves considering the dynamic nature of a system, delays, and changes over time (Forrester, 1994; Richmond, 1993).
3. *Engaging in causal reasoning* involves specifying the relationships between variables and examining the process of constructing relationships and interactions between system components (Meadows, 2008; Stave & Hopper, 2007).
4. *Identifying interconnections and feedback* denotes considering the feedback structures formed by chains that loop back upon themselves, creating circular sequences of cause and effect (Haraldsson, 2004; Richmond, 1993; Sweeney & Sterman, 2000; Zuckerman & Resnick, 2005).
5. *Predicting system behavior based on system structure* requires thinking of a system as a whole (Richmond, 1993; Sweeney & Sterman, 2000), in which the network of relationships between the system’s components interlinks to produce the emergent behavior of the system.

### Students’ Challenges in Applying ST

One of the most notable challenges regarding applying systems thinking to make sense of complex phenomena is students’ inclination to use linear causal chains (Assaraf & Orion, 2005; Grotzer et al., 2013; Jacobson & Wilensky, 2006). Linear causal thinking is characterized by providing explanations in a succession of cause and effect relationships (Perkins & Grotzer, 2005). Though the use of linear causal mechanisms is useful in everyday life and in explaining some phenomena, it is not sufficient to account for mechanisms that often characterize steady states, feedback, cyclic patterns, dynamic relationships, and occasional perturbations (Meadows, 2008), which are necessary to explain complex phenomena such as climate change, the spread of a disease, or a decline in an organism’s population. In particular, the inclination to use linear causal thinking is usually related to immediate direct cause and effect where change in one variable means an immediate change in something else. This stands in contrast with a critical aspect of systems thinking—thinking in terms of change over time and feedback (Grotzer et al., 2013; Richmond, 1993; Tripto et al., 2013). For example, students demonstrate challenges in considering change in processes that take place over a long period of time, such as evolution (Hermann, 2013), or processes that happen quickly, such as reaching a chemical equilibrium (Banerjee, 1991). Furthermore, researchers have described the habits of mind that coincide with linear causal thinking and are at odds with ST aspects. For example, Chi et al. (2012) described students’ tendency to explain phenomena by attributing a central control and deterministic causality element that propels a sequential causal chain of events, which they named “direct causal schema.” However, this line of thinking was not sufficient to explain the underlying mechanism in which various elements interact simultaneously, producing an emergent behavior that differs from each element’s behavior or characteristics (Chi, 2005; Jacobson & Wilensky, 2006; Richmond, 1993). A promising approach to supporting students in applying systems thinking to make sense of complex phenomena is constructing models (Gilissen et al., 2021; Schwarz et al., 2009; Sterman, 2002). In the next section, we elaborate on computational system models and their potential to support students’ engagement in ST.

### Computational System Modeling

Computational system modeling has the potential to support students in learning how to solve thorny problems and make sense of scientific phenomena that relate to complex systems (Stratford et al., 1998; Chandrasekharan & Nersessian, 2015; Sins et al., 2009). The use of computational system models can enable students to explore an interconnected system of multiple variables to explain a phenomenon that learners might otherwise find very difficult to comprehend (Ainsworth, 2008; Linn & Eylon, 2011). The most prominent advantage of computational system models is the ability to manipulate variables to generate model output (Damelin et al., 2017; Schwarz et al., 2007). By running simulations that generate an output, students can compare the model’s output to data available from external resources, such as empirical studies or their own investigations. If data obtained from the model’s output and data from an external source do not match, students can revise their model or question the validity of the data source. Model revision at this point focuses on altering the inputs and relationships between variables. Students can iteratively refine their model, revising their model throughout the modeling process.

Various computational system modeling tools offer different affordances, which result in diverse learning opportunities. In some cases, students use pre-existing models, and in other cases, students construct their own models (Damelin et al., 2017; Mandinach, 1989; Tisue & Wilensky, 2004). We identify two main computational modeling approaches that support the learning of complex systems. The first is agent-based modeling (ABM), in which one makes sense of the system by analyzing interactions between individual constituents of the system, exploring how those interactions result in emergent behavior that is different from the behavior that characterizes the individual constituents (Wilensky & Rand, 2015). The research on ABM has made significant contributions to supporting students in making sense of phenomena and adopting an ST approach (Groeneveld et al., 2017; Jacobson & Wilensky, 2006; Sengupta et al., 2013; Wilensky & Rand, 2015).

The second approach is system dynamics, which is another modeling approach used by scientists to explore phenomena and solve problems. This approach holds promise in supporting student learning. System dynamics models are based on aggregate reasoning in which interactions between system components are considered as stocks and flows (Ossimitz, 2002; Sweeney & Sterman, 2000). Stocks refer to system components that can accumulate or deplete over time, just as containers can fill and empty. Flows refer to the system components that decrease or increase the amount in the container. System dynamic models allow the user to construct nonlinear interactions and structures such as feedback loops and to produce an output that represents system components that change over time (Forrester, 1994; Richmond, 1993, 1994; Sweeney & Sterman, 2000). This approach is beneficial to address two major aspects of systems thinking. The first is the feedback mechanism, which is often necessary to explain the behavior of complex systems (Forrester, 1994; Richmond, 1993, 1994; Sweeney & Sterman, 2000). The concept of feedback can be defined as any action that causes an effect back to the starting point of the action (Haraldsson, 2004). The second addresses a system’s change over time. Many phenomena require the consideration of change over time in which there is a time lag between the cause and effect. In some cases, the delay is negligible, as in certain chemical reactions, and in some cases, the time delay is thousands or millions of years, as in evolution or the formation of a canyon (Assaraf & Orion, 2005; Grotzer, 2003; Kali et al., 2003; Meadows, 2008). Haraldsson (2004) connects thinking in terms of change over time and feedback because feedback necessarily involves time in terms of a time lag. However, human tendency is to think in linear causal patterns and assign agency and responsibility to an event when attempting to make sense of a phenomenon (Galea et al., 2010; Kahneman, 2011; Resnick, 1996). This tendency is at the core of students’ challenges when they come to make sense of phenomena with innate dynamic aspects like erosion, evolution, the spread of disease, and rise in global average temperatures (Sander et al., 2006).

More research should systematically examine the opportunities that a system dynamics modeling tool can offer to students and which challenges still remain. Moreover, there is a lack of description of how such challenges and opportunities align with specific modeling practices.

### Modeling Practices and Their Alignment with ST Aspects

Schwarz et al. (2009) describe scientific modeling as “a process that allows a scientist or a learner to abstract and simplify a system by focusing on key characteristics of a system to explain and predict scientific phenomena” (p. 633), implicitly suggesting that to make sense of a phenomenon, it is helpful to think of it as a system and that a system is the modeling objective. Scholars have described aspects in which the modeling process aligns with and can be informed by ST while regarding ST as an integral cognitive facet of the modeling practice (Forrester, 2007; Sterman, 2002; Weintrop et al., 2016; Wilensky & Reisman, 2006). Previous work (Shin et al., 2022) has further delineated how ST aspects align with modeling practices. This description is congruent with the literature on modeling practices, including the construction, evaluation, revision, and use of a model (Martinez-Moyano & Richardson, 2013; Nunez-Oviedo & Clement, 2019; Schwarz et al., 2009; Sins et al., 2009). It is important to emphasize that the modeling process is dynamic and iterative as students go back and forth between the practices (Pierson et al., 2017; Schwarz et al., 2009). For example, when students define relationships between variables, they might think about the system boundaries differently and, therefore, decide to add or omit a variable, thus constructing and revising simultaneously, or when students simulate a computational model and evaluate its behavior, they may decide to revise their model by defining relationships differently. Next, we briefly describe how the modeling practices align with ST aspects. Because computational models are part of the context of our work, we provide examples for how the aspects are actualized in that context.

#### Constructing the Model

From an ST perspective, it is useful to think of model construction primarily consisting of two modeling practices (Shin et al., 2022): *defining system boundaries* and *designing and constructing model structure*, which for clarity we refer to as *setting relationships* (between model components). Next, we elaborate on each practice.

##### Defining system Boundaries

When constructing computational models to explain a phenomenon or to solve a problem, one should first determine systems’ components whose characteristics are mathematically represented by variables (Arnold & Wade, 2017; Assaraf & Orion, 2005; Stave & Hopper, 2007). Those variables can range across scales, as a foundation for explaining the mechanism that underlies the emergent behavior of the system (Hmelo-Silver, et al., 2017; Levy & Wilensky, 2008). Specifically, in building computational systems models, scientists or students need to identify the input and output variables, defining variables that interact on a particular scale (input variables) and variables that represent the emergent behavior resulting from the network of interactions (output variables) (Arnold & Wade, 2017; Grover & Pea, 2013; Shute et al., 2017; Stave & Hopper, 2007).

##### Setting Relationships

Setting relationships involves specifying how a change in one variable affects one or more variables. This practice allows learners to examine the interconnected nature of the system they model (Meadows, 2008; Stave & Hopper, 2007). Those relationships can vary in complexity, ranging from linear causal chain relationships to nonlinear relationships, including feedback that considers change over time (Assaraf & Orion, 2005; Grotzer et al., 2013). A model’s behavior is determined by the direction of the causal relationships between variables, how the definition of the relationships causes the value of one variable to affect the others, and the overall structure of how the variables are interconnected.

#### Evaluating and Revising the Model

These modeling practices encompass a continual reflection during the computational modeling process, which manifests students’ epistemological assumptions about model construction (Berland et al., 2016; Pierson et al., 2017). Evaluation allows learners the opportunity to run a simulation and manipulate the variables (e.g., increase or decrease the quantity), resulting in an output that shows the effect on all the system’s variables. In general, revising a computational system model is easier than refining physical artifacts or illustrations (Fretz et al., 2002; Bielik et al., 2019; Nguyen & Santagata, 2021). We identify in the literature three observable ST aspects that students use to evaluate their model: (a) identifying how individual cause and effect relationships impact the broader system’s behavior, (b) recognizing how various substructures within a system influence its behavior (e.g., feedback structure), and (c) predicting how specific structural modifications change the dynamics of a system (Richmond, 1993; Sweeney & Sterman, 2000). Table 1 that summarizes these evaluation strategies and their alignment with ST literature provides the rationale for focusing on these strategies.

**Table 1:** Evaluation strategies in computational system modeling and their alignment with ST literature

Evaluation strategy	ST literature
Identifying cause and effect relationships and their impact on the system’s behavior	Exploring multiple cause and effect relationships is a crucial component of systems thinking. To better understand complex phenomena, it is imperative to investigate how variables that do not have a direct impact on each other are interrelated and affect the system as a whole (Shin et al., 2022; Hmelo-Silver et al., 2017; Nguyen & Santagata, 2021)
Recognizing how substructures within a system influence its behavior	Several studies have emphasized the significance of identifying structures within a model and the implications of those structures for the behavior of the system (Haraldsson, 2004; Hmelo-Silver & Azevedo, 2006; Hmelo-Silver et al., 2017). For instance, a circular structure in which a variable initiates a causal chain that loops back to the first variable can indicate the presence of a feedback mechanism
Predicting how structural modifications change the dynamics of a system	Systems thinking involves thinking about change over time and considering a system’s dynamic equilibrium, as well as predicting potential behaviors of the system in response to a shift in that equilibrium (Assaraf et al., 2013; Richmond, 1993; Stave & Hopper, 2007)

#### Using the Model to Explain and Predict

This practice involves the assessment of a model as a method for communicating knowledge. In the context of systems modeling, the system’s behavior expresses the usefulness of the model in explaining the system. The behavior of a system represents the observed system’s attributes. For example, the rate of a chemical reaction is an attribute of the system that results from a set of relationships between various components in the system, such as the concentration of reactants, temperature, pressure, and molecular shape. In computational system models, students can assess whether their model, which includes a network of interconnected relationships between variables, results in expected system behavior that is able to explain various conditions of the system and predict what happens in the case of a perturbation to the system. The assessment involves comparing the model’s output to other existing models or external data, articulating the differences between the model and the underlying real-world phenomenon and considering the limitations of their model (Schwarz et al., 2009). Specifically, some system models are used to describe and predict the behavior of a system over time, which renders a consideration of the dynamic nature of a system and its changes over time (Forrester, 1994; Keynan et al., 2014; Richmond, 1993).

## Research Questions

This study aims to delineate the opportunities and challenges students encounter in applying ST aspects while modeling in a system dynamics approach to make sense of a phenomenon. Therefore, our main research question is: What are the opportunities and challenges students experience when constructing and using system dynamics models to make sense of a phenomenon? We focus on how students apply ST when using different modeling practices in the context of system dynamics modeling: constructing, evaluating, revising, and using the model**.**

## Methodology

### Development of Project-Based Learning-Aligned Curriculum Materials

A chemistry unit based on project-based learning (PBL) principles (Krajcik & Blumenfeld, 2006) was co-designed by classroom teachers and the authors of this paper. The unit aligns with the Next Generation Science Standards (NGSS Lead States, 2013) high school performance expectation HS-PS1-5 – Apply scientific principles and evidence to provide an explanation about the effects of changing the temperature or concentration of the reacting particles on the rate at which a reaction occurs. The unit focused on the kinetics of chemical reactions and consisted of five 80-min lesson blocks over 2 and a half weeks of classroom instruction (Bain & Towns, 2016). Before students started the unit, they discussed the purpose of building models and had hands-on experience with a system dynamics software called SageModeler for 4 h. SageModeler is a web-based open-source tool designed to support student learning through constructing, evaluating, revising, and using models (Bielik et al., 2018, 2020). Students used the dynamic time-based setting in SageModeler, which utilizes a “stock and flow” system dynamics approach (Zuckerman & Resnick, 2005).

Students were introduced to the software and its basic functions and built models to explain phenomena about simple dynamic everyday life scenarios (e.g., bathtub water levels, money in the bank). At the beginning of the chemical kinetics curriculum unit, students were presented with a scenario of a stain on a shirt and a bleach pen that could not remove the stain. The driving question was, “What can you do to speed up the removal of a stain?” The anchoring phenomenon was dissolved food coloring that gradually fades once bleach is added. At the beginning of the unit, students added their own questions about the phenomenon using a driving question board, which was addressed throughout the unit (Weizman et al., 2008). The unit introduced students to three key scientific principles related to the disciplinary core ideas found in the *Framework* (NRC, 2012): (1) reactions can occur due to a collision between molecules (i.e., reactants), (2) an increase in temperature increases the frequency and force of these collisions, thereby increasing the reaction rate, and (3) higher concentrations of the reactants will result in an increased frequency of collisions, thereby increasing the rate of reaction. Initial conditions of the chemical system can be used to identify its emerging properties, specifically how changes in the initial conditions of reactants in a chemical reaction affect the rate of a reaction over time.

The scientific principles outlined above pertain to systems thinking concepts explored in this study. Properly defining the boundaries of the system is critical when investigating chemical reactions. To understand the phenomenon in question, it is necessary to differentiate between reactants and products and to identify key variables such as temperature and concentration that impact the rate at which reactants are converted to products. Recognizing the rate of reaction as a crucial output variable that reflects the macroscopic behavior of the system is essential for comprehending the phenomenon and answering the curriculum unit’s driving question.

Addressing the reinforcing feedback mechanism that characterizes the chemical reaction between bleach and dye is crucial for understanding the behavior of the reaction rate over time. As the chances of collision between reactant molecules in the system increase, so does the likelihood of product formation, which consequently reduces the concentration of reactants, and ultimately diminishes the possibility of additional collisions between reactants that result in product formation. This reduction is observed as a decrease in the rate of reaction.

Given that the phenomenon focuses on the rate of reaction, which represents the change in reactants and products over a unit of time, it is necessary to think in terms of change of the system’s components over time to explain the phenomenon under investigation.

The unit was administered through an online activity system, which embedded the SageModeler software. It also included science demonstration videos, simulations of particle behavior, and questions that aimed to help students construct, evaluate, revise, and use system models over four model revisions. Students were explicitly and often reminded that the goal of using SageModeler was to create a model that supports them in answering the driving question. Most students worked in pairs with a few groups of three as they were sitting next to each other sharing one computer screen. The teacher walked around the class using probing questions and answering students’ questions. One of the researchers who attended the classes supported any technical issues that came up. The unit was arranged in a way that after approximately 20 min of student work on the computer, the teacher gathered students for a plenary discussion about what was learned so far that addressed questions on the driving question board and invited students to add new questions as the learning progressed.

Throughout the unit, students collected and analyzed data that drove the revision of their models. The data could be experimental, or based on a simulation, table, or graph. They explored various factors that might affect reaction rate, including the temperature and concentrations of reactants. Important to note is a hands-on experience in which students collected and analyzed real-world data using a spectrophotometer, entering their experimental data in a table in SageModeler, allowing comparison between graphs generated as the model’s output to those generated from experimental data. The activity was designed to provide students with the means to comprehend the emergent properties associated with each of the three key scientific ideas. Students conducted a number of experiments in which they kept a constant concentration of the reactants with a varied temperature of the solutions and alternatively a constant temperature with varied concentration. This activity also allowed students to generate and analyze graphs that feature exponential reductions in absorbance (a measure of unreacted dye) over time and provided them with an opportunity to visualize the decrease in the rate of reaction over time. Detailed descriptions of the unit and more information on how the phenomenon is represented in SageModeler can be found in Appendix 1.

### SageModeler Features That Support Aspects of ST

Time-based models are often challenging for students because they require consideration of changes in a system over time and include aspects that depend on feedback from interconnected and potentially rate-limiting factors (Sweeney & Sterman, 2000; Tadesse & Davidsen, 2019). SageModeler’s dynamic time-based models serve as a promising platform to investigate in more depth students’ engagement in ST aspects like feedback and change over time. As a computational model, it generates outputs, which can be represented as a graph showing a change of a designated variable over time. SageModeler also allows the ability to set causal relationships between designated variables, including the directionality (increase or decrease) and the magnitude, which is represented by words and an accompanying graph (“about the same” is a linear graph, “more and more” is a logarithmic graph, and “less and less” is an exponential graph). Another promising feature of SageModeler is that it allows learners to import real-world and experimental data or output from other expert models and compare it to simulation output generated from SageModeler. Learners can then create graphs from these various data sources and make decisions about the validity of their model. This functionality is essential for several reasons. First, from an epistemological standpoint, this process allows learners to experience how computational models help scientists make sense of the world. Second, revising models based on new data allows learners to experience the tentative nature of models, thus strengthening their metamodeling knowledge (Fortus et al., 2016; Schwarz & White, 2005). Finally, comparing real-world or experimental data and the output generated by the model supports learner reasoning and sensemaking (Schwarz et al., 2009). As mentioned in the previous section, we utilized this unique affordance by allowing students to compare computer simulation and experimental data with their models. For example, SageModeler automatically generated graphs from the uploaded experimental data, allowing the comparison between the graphs generated by the model students constructed and the experimental data. Additional information about SageModeler and the way its features align with the modeling process can be found in Appendix 2.

### Participants

In the spring of 2019, the chemistry unit was enacted in five high school classes, taught by two science teachers, Mr. H. and Mr. M., and included 100 students. The high school was a US Midwestern STEM charter school with an accelerated STEM learning program. Students accepted to the charter school came from 16 school districts. We selected eight 10th grade students (seven female, one male) who completed the unit activities in pairs. Two pairs from each teacher (Groups 1–4) served as four participant groups, chosen based on our request to teachers to recruit students who tend to be more talkative and verbalize their thinking. The students varied with respect to individual academic achievements and backgrounds and are thus a representative sample of students in the classes that took part in the intervention. Although both teachers were experienced, they had different characteristics. Mr. H. had 15 years of teaching experience, a master’s degree in chemistry, and teaching certifications in chemistry and psychology. Mr. M. had 4 years of teaching experience, a bachelor’s degree in zoology, and teaching certifications in chemistry and biology. The teachers participated in 10 h of face-to-face and remote professional learning via videoconference focused on how to support students in using SageModeler to model various phenomena. As part of this support, the authors familiarized teachers with the modeling process, practices, and ST jargon. Additionally, the authors conducted a reflective discussion with the teachers after each lesson.

### Data Sources and Analysis

Some of the data that support the findings of this study are available in the supplementary material in the Appendix. Other data that support the findings of this research are on request from the corresponding author. The data are not publicly available due to containing information that could compromise research participants’ privacy/consent.

#### Student Models

Students constructed and revised SageModeler models in groups. Most groups had two students, with a few groups having three students. They saved their models to an online learning platform that allowed researchers to collect and examine their models. The curriculum was planned for students to complete four model revisions. We analyzed and scored the models using a quantitative rubric targeting two of the three modeling practices described above: *defining system boundaries* and *setting relationships*, which are both part of *constructing* and *revising a model* and *using the model to explain and predict*. The rubric was not used to assess the *evaluating and revising* modeling practice. We used screencasting software, which we describe in more detail below, to assess the *evaluating and revising* practice as students’ models do not provide much indication about the deliberation that took place during the iterative refinement process (Shin et al., 2022). Our goal was to indirectly measure student use of ST through the scoring of modeling practices, given our previous proposition that each practice involves specific aspects of ST. Due to its length, the rubric and the scoring method can be found in Appendix 3.

The validity of the rubric was established by four internal scholars who have an expertise in modeling and systems thinking. The researchers were separately asked what modeling practice and ST aspect they think each item in the rubric is designed to evaluate and whether they think it does. The researchers had a consensus on all the items. In addition, the rubric was sent to an advisory board that included experts in systems thinking and modeling. None of the board members reported any issue with the rubric.

To measure reliability, the researchers thoroughly discussed the scoring criteria, independently scored the fourth model revision (fifth model), compared scores, and reached a consensus. Only 8 of the 47 groups of students completed the fourth model revision. We started with the fourth model revision as it likely represented the most refined version of the students’ knowledge; the richness of the models allowed us to flesh out additional issues and potential disagreements between the scorers. The researchers reached an initial 90% agreement on the models’ evaluation and, after further discussion, reached full agreement. Next, two researchers independently scored the rest of the model revisions of all groups, reviewed each other’s scoring, and reached a full consensus after discussion about specific disagreements.

However, because most students did not get to their fourth model revision because of classroom time limitations, we decided to exclude the eight models of the groups who reached the fourth revision. Furthermore, we found almost no significant differences between the third and fourth revisions. Therefore, in the results, we follow students’ modeling progression at four time points: initial model, first revision, second revision, and final model (third revision).

#### Student Responses on the Learning Platform

The chemical kinetics unit was administered through an interactive learning environment where students could enter their answers to various questions during the learning process. We analyzed student responses to two questions, one related to the mechanism of the phenomenon and the second related to the evaluation of the model using real-world data. Both questions were just prior to students’ final model revision (third revision). The first question was: “Explain, at a microscopic level, how the absorbance of the sample is affected over time.” We sorted the quality of the answers into seven levels. Students were asked to answer this question after they completed a hands-on experiment and obtained results from the spectrophotometer. The levels were based on Grotzer’s work on dimensions of causality (2003). The differences between the levels are based on a shift from a linear causal explanation to an explanation that considers change over time and feedback. The criteria for each level with students’ examples are shown in Table 2. Three authors reached an 85% and 100% inter-reliability agreement before and after discussion, respectively, on 47 cases (Kappa value 0.84, *P* < 0.001).

**Table 2:** Levels of students’ causal mechanism answers of the phenomenon and sample responses

	Definition	Sample student response
Level 0	No answer	
Level 1	No relevant explanation. The explanation is not relevant to addressing the mechanism for the drop in absorbance over time OR Erroneous explanation. The explanation does not agree with a canonical science explanation	“When the molecules become more basic, the particles’ ability to absorb over time decreases. The bleach made the solution more basic.”
Level 2	A causes B explanation, in which the drop of absorbance over time is explained by a simple linear cause and effect that does not refer to the particle level	“The absorbance of the solution decreases due to the increase in clear water.”
Level 3	A causes B causes C explanation, in which the drop of absorbance over time is explained by a linear causal chain. The impact of each variable on the other seems to be instantaneous	“The bleach breaks apart the red dye, decreasing the number of dye particles and making the solution more transparent. This causes it to absorb less of the light passing through it, meaning the absorbance has decreased.”
Level 4	A causes B explanation, which addresses a **gradual** change over time. Students attribute the gradual change in absorbance to the change in the amount of reactants and products over time	“As the bleach lightens the color more and more, the absorbance of light will decrease because there are less dye particles.”
Level 5	A causes B causes C explanation, which addresses a **gradual** change over time. Students provide a causal chain in which there is at least one relationship between three elements, and attribute the gradual change in absorbance to the change in the amount of reactants and products over time	“As the water becomes clearer because the bleach breaks up the bonds that make the red dye red, the solution has less red dye so there's less red dye to absorb light. Therefore the absorbance of the solution decreases over time.”
Level 6	Explanation that addresses the **gradual** change over time and includes **feedback**. Students provide a causal chain that loops back on itself. A possible explanation addresses the collisions between particles’ reactants that causes an increase in the amount of products, which in turn reduces the chance for collisions between reactants that can yield a product and hence decreases the reaction rate	No examples were found

The second question was: “Compare the graphs generated by your model and those that indicate the experiment results. Notice that the graphs indicate time as the independent variable and absorbance as the dependent variable. Do you notice any difference in the trend line between the graphs? If so, describe it.”

The rubric for evaluating the second question appears in Table 3. Three of the authors reached an 84% and 95% inter-reliability agreement before and after discussion, respectively, on 20 cases (Kappa value 0.694, *P* < 0.001). The significant drop in the number of cases is due to the fact that the teachers increased the pace towards the end of the unit as they felt pressure to cover other curriculum goals, resulting in many groups of students not completing the tasks that asked them to compare their model output to their experiment results. Thus, we did not include level zero in the rubric for those who did not answer the question.

**Table 3:** Level of students’ description comparing model output to real-world data and sample responses

Level	Description	Sample student response
1	The description only pertains to either the model or the experimental data without any comparison between the two. The students have not addressed the differences or similarities between the output of the model and the experiment	“The slope of the trendline on the graphs collected from the model will decrease as the temperature increases.”
2	The description covers both the model output and experimental data, but the students only focused on the trend exhibited in both graphs. They have not described any differences or similarities in the patterns shown in the model output and experimental data	“The graphs decrease”
3	The description pertains to both the model output and experimental data, and the students have addressed some differences between the two. However, these differences are esoteric. The students have not addressed any similarities or differences in the trend exhibited by both the model and experimental data over time	“Yes, since only one trial of each factor is being experimented with there are not three trend lines per graph in the model graphs. The graphs for the sage model are way higher in absorbance than the graphs from the data points.”
4	The description pertains to both the model output and experimental data. The students have identified differences between the two by noting the variation in the behavior of the dependent variable over time	“Yes, the simulated graph decreases at a much higher rate.”
5	The description covers both the model output and experimental data. The students have identified differences between the two by comparing the magnitude represented in the graph of each output, such as a linear versus exponential trend, as well as the behavior of the dependent variable over time. They have noted how these differences impact the interpretation of the data and any conclusions that can be drawn from them	“Yes, the simulation has a linear slope until the very end when it levels out while the real data increases at a decreasing rate.”

#### Screencasts

A screencast video simultaneously captures the actions students perform on screen including constructing their models as well as students’ voices using the computer’s microphone. The screencasts were recorded on classroom laptops and documented students’ interactions with the system modeling software and discussions between students as they constructed, evaluated, revised, and used their models. Screencasts were recorded each time students used the modeling software. Approximately three screencasts were recorded for each participant group, each 60–70 min long. The total time dedicated to modeling was 100–120 min.

We used screencasts for two main reasons. First, we wanted to capture students’ discussions to characterize the modeling practice of *evaluating and revising.* Second, we wanted to determine how students’ progress in evaluating and revising their models affects other modeling practices and their understanding of the phenomenon. The analysis was event-based, in which we looked at all student screencasts for specific events that demonstrate modeling practices and the operationalization of ST aspects. We identified events in which students performed model simulations and reflected aloud on the outcomes of the model behavior. An event was identified as an *evaluating and revising* episode if it met all of the following criteria: (1) students clicked the “Simulate” button in SageModeler, (2) students moved a slider that controlled at least one variable, and (3) students verbalized their intentions with statements such as “So, what do you think?” “That works,” or “I do not understand what’s going on.” The screencasts also allowed us to follow teachers’ support that was given to the students who volunteered to take the screencast because we could hear them through the microphone.

We used ATLAS.ti 8 software to capture and mark the episodes. Two of the authors analyzed the screencasts. The authors independently identified and analyzed the *evaluating and revising* events. A comparison showed a complete agreement on the episodes in each model revision. Following this, the two authors analyzed those episodes, identifying students’ reasons for simulating the model, whether it was based on their own decision or initiated following a teacher’s suggestion. In addition, students’ reflections and software moves after simulating were followed. We defined teacher-prompted *evaluating and revising* events as ones in which the teacher explicitly told the students to test their model or to click the “Simulate” button. The prompts were obtained from an analysis of teacher-student interactions. The average length of such interaction was 3 min.

#### Student Interviews

Semi-structured interviews were conducted 2 weeks after the unit’s completion with four students, one from each case. Based on the teachers’ descriptions, the students moved on to learn a different concept in chemistry that was not related to kinetics. Each interview lasted approximately 30 min. During the interviews, students were asked to describe their thinking processes and strategies when building their final model, to explain how the model helped them answer the driving question, and to share their overall experiences with the unit. A full transcription of the interview protocol can be found in Appendix 4. Interviews were recorded and fully transcribed. We analyzed the interviews using a set of codes, sorting the codes into categories, and defining relationships and patterns between the categories (Saldaña, 2021). We used ATLAS.ti 8 software for analyzing the interviews. We based our primary coding on the four modeling practices (Schwarz et al., 2009) and prominent aspects of ST like thinking in terms of change over time, feedback mechanism, and thinking across scales. We also coded the interviews based on the key scientific ideas of the phenomenon. We searched for patterns in the data that examine the interconnection between modeling practices, ST aspects, and students’ use of key ideas to make sense of the phenomenon. We also looked for challenges in technical issues or user interface questions in addition to challenges that relate to ST. Additional categories emerged during the analysis of the interview data that relate to students’ epistemology about models, collaboration, interest, and motivation.

## Results

We divide the results into two parts. In the first, we describe the results of students’ progression in the *setting relationships* modeling practice throughout the unit. In the second part, we detail the opportunities and challenges students encountered when attempting to construct a time-based model and the reasons that led to a shift from a linear causal explanation to an explanation that considers change over time and feedback. This analysis revealed two emerging themes. The first theme is related to the opportunities and challenges students’ face in constructing dynamic time-based models. The second theme is related to opportunities and challenges students face using real-world data when evaluating and revising their models.

### Students’ Progression in the *Setting Relationship* Practice

**Fig. 1:**
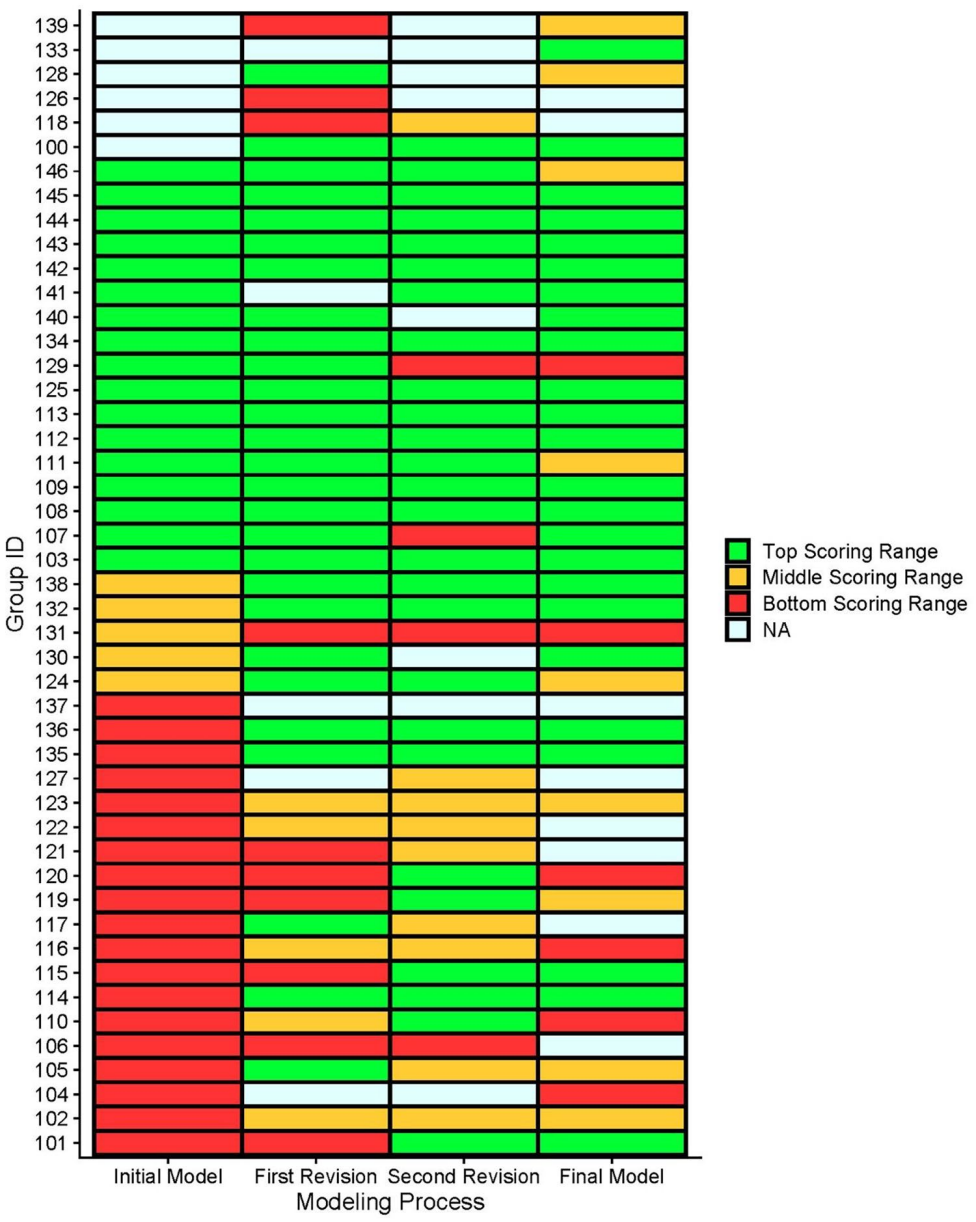
Group scores for “setting relationships” practice at four time points. Different colors represent the three scoring ranges as shown. “NA” indicates that the students did not complete a model or that the model was not saved due to technical issues

Figure 1 shows the scoring range of students’ models related to the *setting relationships* practice at different time points in the unit (see Appendix 3 for the rubric). We defined three scoring ranges: top, middle, and bottom. In particular, we were interested in the points in which students’ scores increased or decreased between revisions. After comparing the models before and after a shift in the level of *setting relationships modeling* practice, our analysis showed that scores increased when students revised their model from a linear causal chain representation to a dynamic one (e.g., by adding collectors and transfer valves). That means students set relationships that properly represented the dynamic mechanism of the phenomenon. In this case, students represented the relationship between the amount of reactants and the amount of products as a transfer relationship rather than in terms of a causal relationship.

Also, we found that the main reason that hindered students in making progress in the modeling process was setting the relationship between variables in a linear causal chain. Moving to a more sophisticated relationship patterns resulted in improvement. We can identify those cases of improvement, especially between the initial model and the second revision. For example, Groups 124, 130, 136, and 137 all showed improvements that resulted in models with a dynamic representation of transfer in which reactants turn into products.

### Students’ Opportunities and Challenges

In this part, we identify two major themes. The first theme addresses opportunities and challenges in constructing dynamic models. The second theme addresses opportunities and challenges in using real-world data in evaluating and revising models. Each theme is further divided into two sections.

#### Theme 1: Opportunities and Challenges in Constructing Dynamic Time-Based Models

This theme is divided into two sections. The first focuses on students’ challenges in representing reaction rate; the second shows how the simulation feature of the modeling software and teachers’ prompts both support students in evaluating and revising their model. Because the unit focuses on chemical kinetics, we were interested in determining how students defined changes in the reactants and products over time and how they represented the factors that affect reaction rate. To recognize time as an essential component of the system, students needed to make sense of the rate of chemical reactions, but we found that students had difficulty conceptualizing rate in the context of the phenomenon.

##### Representing Reaction Rates

All four participant groups included a “time” node in their initial models. By “time” students meant the “end of reaction” or “reaction completion” as the common terms used in the classroom to describe the time it takes for the reaction to reach equilibrium. (Students did not study and had limited knowledge of the concept of equilibrium when the unit took place.) It is important to note that time is part of the model simulation’s output that shows the change of a certain variable over time, rendering time as an unnecessary variable to model this scientific phenomenon.

**Fig. 2:**
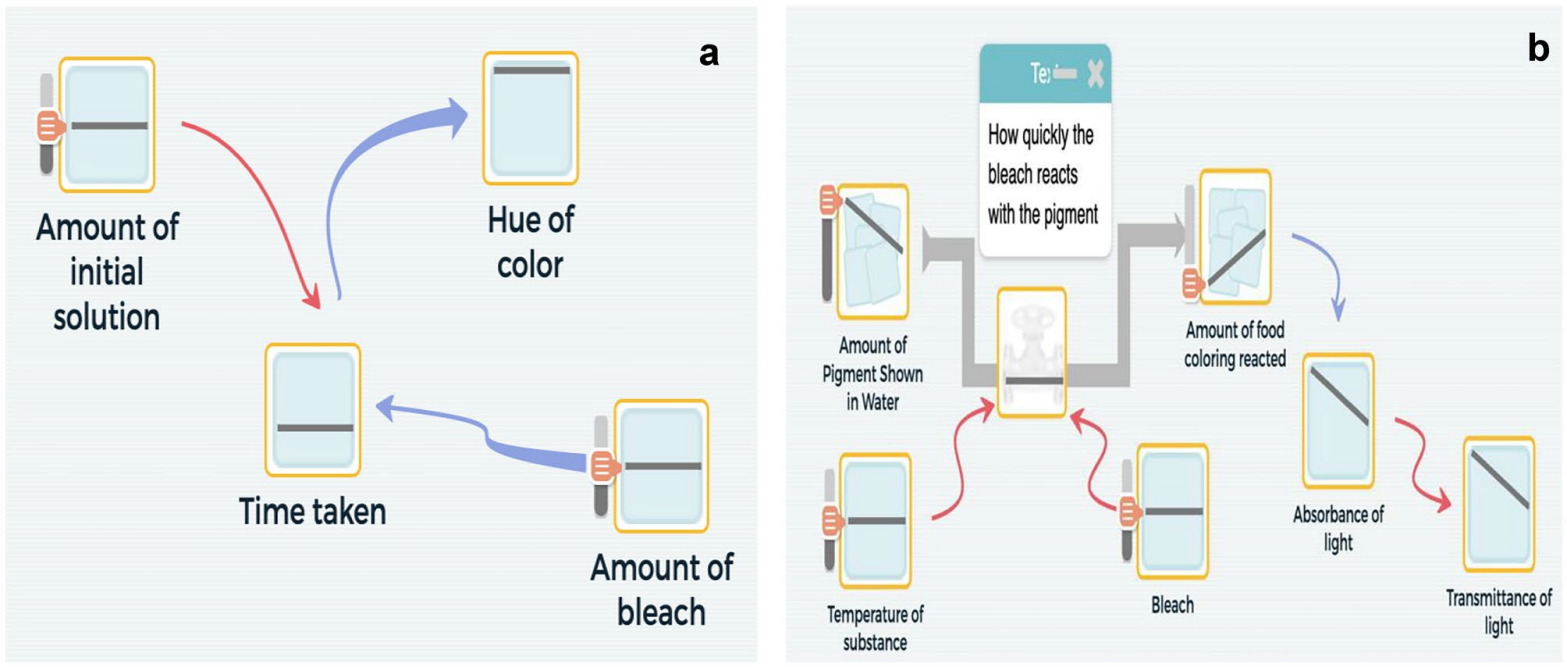
Sample student models. **a** A model that represents no change over time (Group 2; initial model). **b** A model that represents change over time (Group 1; second revision). In each model, the “mini-graph” within each node shows the variable’s value over time

Moreover, including time as a separate variable in the system affected students’ line of reasoning and the relationships they set in their models. Group 2’s model serves as an example of the linear causal line of reasoning that characterizes students’ initial attempts (Fig. 2a). Following the line of reasoning in the model, the initial concentration of reactants (represented by the amount of initial solution, which is the red dye and bleach) affects the time it takes for the reaction to reach equilibrium (represented in the model as “time taken”), and “time taken” affects the concentration of the products. The first relationship shows an appropriate relationship that ties the concentration of reactants with the “time taken” variable. However, the relationship in which “time taken” effects the “hue of color” positions time as a component that affects the products of the reaction. This line of thinking is analogous to thinking that time is a factor that affects the healing of a wound, when it is the various factors that come into play that affect how long it takes for a wound to heal.

**Fig. 3:**
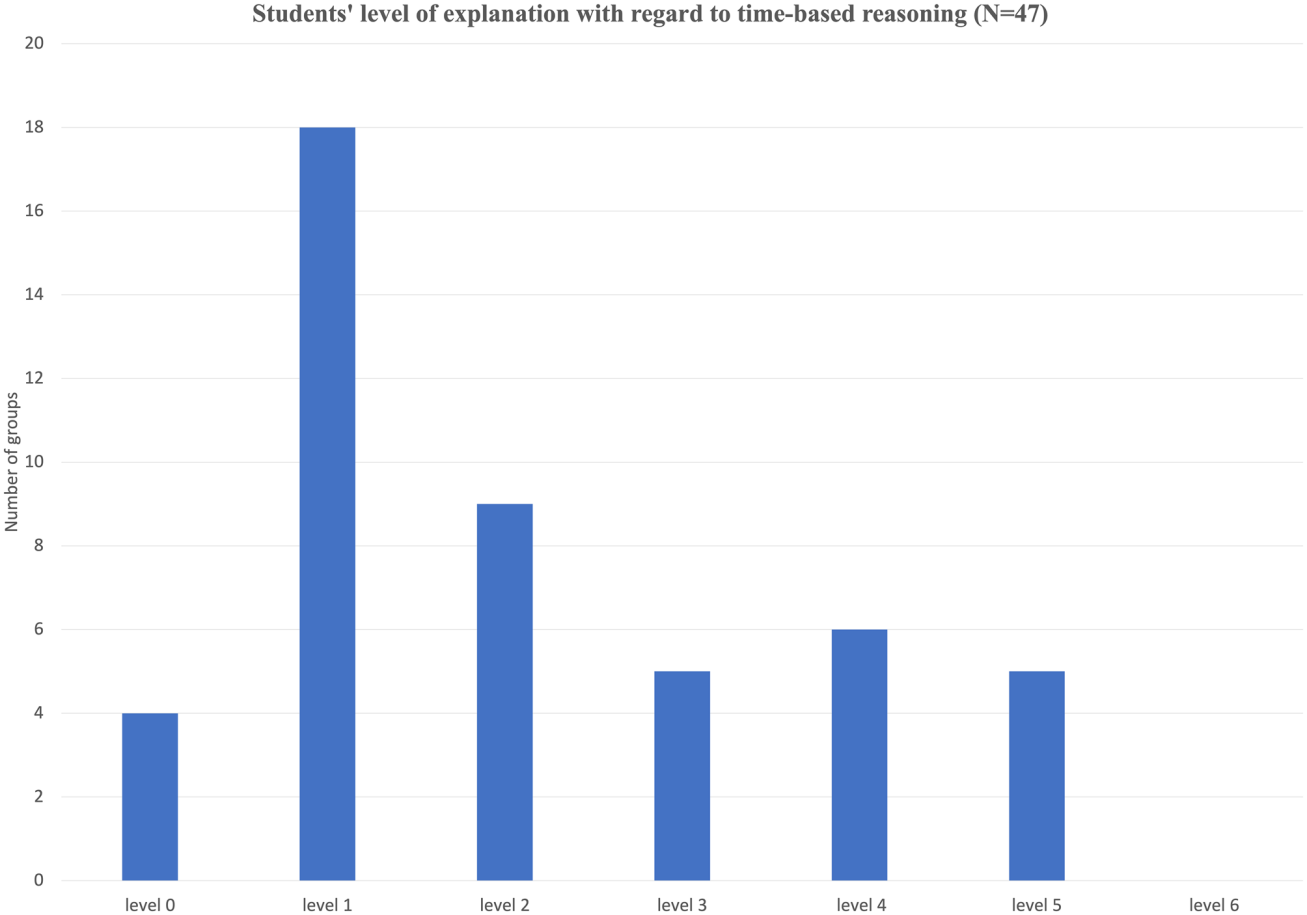
Distribution of students according to the level at which they addressed change over time in their explanation. (See Table 1 for a description of the levels.)

In addition, we paid close attention to students’ discussion of dynamic aspects while building their initial models. For all groups, the prevailing line of thinking regarding time was linear causal thinking. For example, Group 3 observed: “The red dye didn’t clear as fast as in the beginning (of the reaction).” Noticing that the reaction goes slower over time could have led to thinking about the mechanism that leads to the changes in the reaction rate over time; however, during their discussion, the students explained the phenomenon by following a causal chain of reasoning: “The amount of bleach affects the amount of time taken for the dye to fade, which affects how much red dye pigment is left.” These results show that the way students addressed time in their model affected their linear causal description and mechanism of the phenomenon. Furthermore, an analysis of students’ descriptions of the mechanism of the phenomenon reveals that 18 groups did not understand how absorbance is related to the rate of the chemical reaction. The most prevalent erroneous explanation is that the substance has a capacity to absorb light, and this capacity decreases over time. The rest of the groups used a linear causal relationship at different complexity levels with none addressing feedback mechanisms (Fig. 3).

Despite the challenges mentioned above, we found that most students eventually revised their model to include dynamic features, and the revisions elicited students’ use of time-based terminology to explain their models. Three of the four participant groups (all except Group 2) expressed ideas that emphasized the dynamic properties of the phenomenon as they explained their model during the interviews. Those groups used words or phrases that included “During this…that happens, over time, and as time goes by” to explain the mechanism of the phenomenon.

We further examined possible reasons that led to student groups deleting any variables related to “time,” identifying two main reasons for students’ revision of their models: simulating their models and teacher support.

##### Running a Simulation and Teacher Support Prompt Model Revision

The screencasts show that running simulations led the students in Group 1 to conclude that the time variable did not contribute to their model, and therefore, they decided to delete it. The following quote from a student in Group 1 describes how the simulation feature and teacher support helped them to make sense of the phenomenon.

“Well, we pretty much just decided it. Trial and error. We had these connected here at one point and this connected to this [pointing at the screen]. Because at first, we didn’t have this valve [a symbol for a dynamic relationship] thing here. We had just a normal relationship. So, just trial and error, and conversations with Mr. H.”

When the student was asked what they meant by “trial and error,” they replied they ran a simulation of their model and moved the sliders of various variables to see if they matched the expected behavior.

However, conducting simulations (which we interpret as a sign of evaluating the model) was not as intuitive as we thought it would be for other groups. Examining the screencasts of students’ first attempts to run simulations using their model revealed that Groups 2 and 3 interpreted the mini-graphs as a simplified bar graph and not as a representation of a variable’s change over time. Figure 2 presents examples of two model simulations. Figure 2a shows a simulation from Group 2’s initial model, where one can observe no change over time as all the graphs inside the nodes show a horizontal straight line, while Fig. 2b shows a simulation from Group 1’s second revision in which one can observe linear change over time. The challenge in understanding that time is integrated and computed as part of the modeling output caused students to misinterpret the model output. In most cases, students required the teacher’s intervention with a suggestion or a hint to simulate the model. Table 4 shows the number of simulations students initiated and those prompted by the teacher.

**Table 4:** Number of simulations performed during Lessons 1 and 2 in the chemical kinetics unit

**Group #**	**Lesson 1** **(Initial model construction)**		**Lesson 2** **(First and second model revision)**	
	Student-driven	Teacher-driven	Student-driven	Teacher-driven
1	6	0	5	0
2	0	2	3	2
3	0	2	2	0
4	3	2	3	0

As the teachers helped students make sense of the model output, they also supported students in shifting to a time-based model representation and reasoning. For example, Group 3, which made the most significant improvement moving to self-initiated simulations, started with a linear causal chain representation of the phenomenon, then changed their representation and way of thinking after the teacher’s intervention. The teacher encouraged the students to simulate their model and pay attention to the model output. Here are some quotes of prompts from both teachers:

“Are you looking at a scenario that is changing over time or is it static?”

“What is changing and how could you see it in the model?”

“What does the x-axis of the graph represent?”

Those prompts led the students to propose that “time is just there,” meaning it does not influence other variables but that the reactants and products change over time.

#### Theme 2: Opportunities and Challenges in Using Real-World Data to Evaluate and Revise Models

As mentioned, students had the opportunity to compare their model with real-world experimental data as an opportunity to link the system’s behavior and its underlying feedback mechanism. We assumed that when students compared the experimental data and the model output and noticed any mismatch between the two that students would be encouraged to revise their model, which would lead to reconsideration or addition of relationships to the existing model. As described above, students collected data on the solution’s absorbance with red dye and bleach over time in different concentrations and temperatures, using this data to generate a graph of absorbance over time. The curriculum design is intended to motivate students to consider the connection between the behavior of the system and its underlying feedback mechanism. Although we did find that students used this opportunity to compare real-world data with the model output, students faced significant challenges in using the data as a source for a meaningful model revision.

This theme is divided into two sections. The first section discusses the characteristics of the revision that follows the comparison of the model to real-world data. The second section describes students’ epistemological assumptions regarding the use of real-world data.

##### Characterizing Model Revisions That Followed Comparison to Real-World Data

**Fig. 4:**
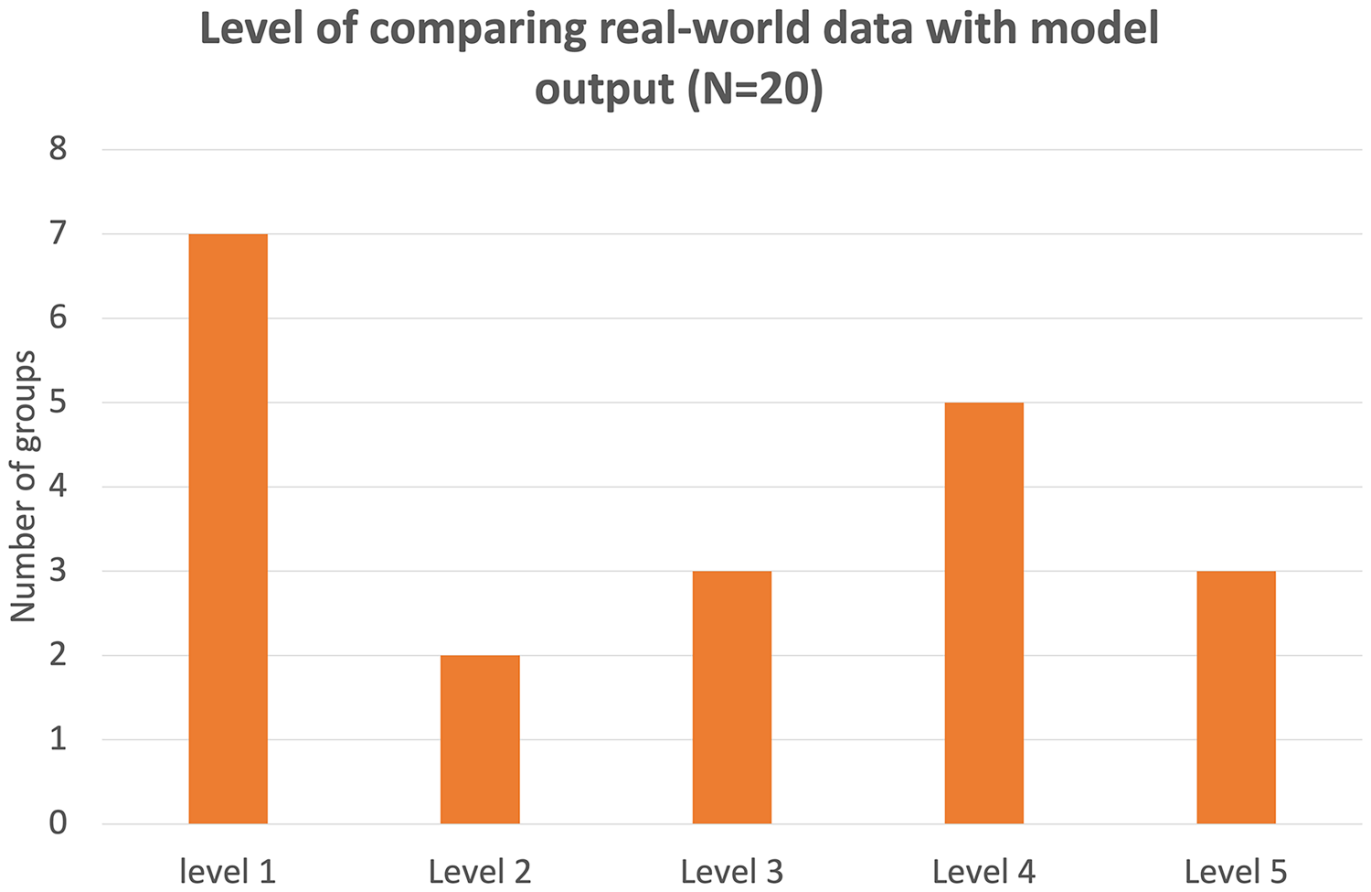
Distribution of students based on the level of their comparison between real-world data and model output (see Table 2 for description of levels)

Figure 4 shows that 7 groups (14 students) who answered the question that asked them to compare their model output with the experimental data did not do so. Only three groups reached Level 5, noticing the difference in the data generated from the models and the experimental data (i.e., that the output from the model shows a linear trend while the experimental data shows an exponential decrease over time). It is important to note that most students were able to compare the model output and the experimental data to various extents (Levels 2–5).

The quantitative analysis matches the results observed in the participant groups. All the participant groups noticed the difference between their model and the experimental results. The most common strategy to make the two outputs match was a trial-and-error approach in which students tried to change their model so the behavior of the output variable (absorbance) would fit the behavior of the experimental results. Groups 1, 3, and 4, all of whom had a dynamic representation of the phenomenon by the time they generated their experimental data, tried to achieve the same behavior by modifying the variables that were directly linked to the level of absorbance instead of examining the net of all relationships between variables in the model. The software allows one to set the magnitude of the relationship between variables, which is also represented by an accompanying graph. Students’ initial approach was to set a direct relationship between variables, typically concentration of products to level of absorbance. The following provides an example of setting such a relationship in SageModeler: “an increase in the concentration of products causes absorbance to decrease by less and less” (an accompanying graph shows an exponential decay). The logic that seemed to guide the students is whether the relationship graph resembled the experimental result graph.

Once that approach did not yield the expected behavior, those groups tried different relationship settings using a trial and error approach, yet all were focused on a direct relationship with the absorbance variable. In this case, student groups did not discuss the mechanism that underlies the emergent behavior that causes absorbance to behave the way it does. In those trials, the underlying assumption is that the change in behavior will result in a direct relationship and not in a network of relationships.

**Fig. 5:**
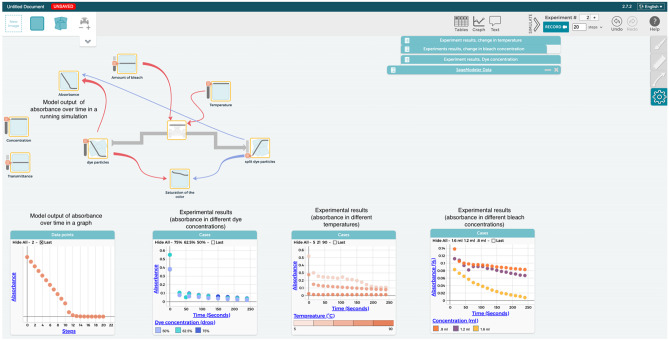
A screenshot taken from Group’s 3 model showing graphs of simulation results next to graphs of experimental results. The model behavior is represented both in a graph and inside the nodes, which generated a linear decay of absorbance. The experimental results show an exponential decay of absorbance over time as concentration of the reactants and temperature of the solution change

Groups 3 and 4 reached a model behavior that matched the experimental results’ trend (absorbance decreases over time) but not the exact behavior (exponential decay). Those students were satisfied with their effort, saying, “it looks more or less the same” and “good enough.” Fig. 5 shows the comparison that Group 3 made between the model output and experimental data. Group 1 model had a similar trend as Groups 3 and 4, but the students were not satisfied with the results and continued trying to achieve a more accurate matching.

Unfortunately, the teachers were not responsive to the students’ challenges at that point of the unit and hurried to finish the unit with a brief summary that did not tie the change in absorbance over time to the mechanism that underlies this change.

Epistemological Assumptions About Using Real-World Data.

The interviews provided additional information about students’ reasoning as they used real-world data to revise their model and address feedback. In the interview, a student from Group 1 said that they noted the differences between their model and the experimental data, but reported they could not get the model to behave the same as the experimental data. The following quote describes the challenges students faced in representing the mechanism of the phenomenon, which partly relates to the need to consider the change in reaction rate over time within the whole system.

“So, that was saying that the transmittance was going up and the reaction was going faster. That was just saying that the reaction was happening really fast and then it was slowing down...I think it’s just confusing to me. We set it up and it made sense with every step but looking at it as a whole it’s confusing.”

The interviewed student from Group 3 attributed the difference between the model and experimental data behavior to their lack of proficiency with the experimental procedure, even though their data collection was performed appropriately. The student said they did not conduct more changes to fit the model to the results because they believed that the computer model was more accurate than their experimental results, as described in the following quote.

“Well, with the model, it is going to be a perfect scenario. Obviously, the data that we collected isn’t perfect because that’s just not how it works. It is as close as we could, but it couldn’t be exactly like the model. There are no outside factors affecting it [the model], other that [than what] you put into it.”

Only students from Groups 1 and 3 were able to address the dynamic nature and feedback of the phenomenon verbally, as demonstrated in the following quotes:

Student from Group 1:

“Yes. So, it’s decreasing at a decreasing rate (the rate of reaction). At first, there are a lot of particles in there (the test tube) reacting with each other and then over time it just gets less and less. But they’re still reacting with each other. It just takes more time for them all to react.”

Student from Group 3:

“Initially, when you’re first introducing the bleach to the dye there’s a greater chance of the particles colliding because none of them have reacted with each other yet. Then as you go on, not as many of them still need to react so it takes time for them to actually react because of the bleach.”

In both examples, the students provide a mechanism that explains the decrease in the rate of reaction over time. They regard the time delay, which is explained by the dropping concentration of reactant, as resulting in more time to have a collision that results in a product. The difference between the students is that the student from Group 3 did not link the behavior (graph of change in absorbance over time) to the feedback mechanism because she perceived the real-world data as untrustworthy. Despite reaching the highest level of understanding of the phenomenon, the epistemological stance of Group 3 students blocked a possible link between the mechanism and real-world data.

It is interesting to note that those explanations came before students walked the interviewer through their model. Similar to students from the two other groups, they stated the key ideas of the phenomenon in a cause and effect fashion, showing a tendency to default to linear causal explanations. Here is a quote from a Group 1 student who displays this thinking.

“If bleach increases and the amount of pigment increases, then that’ll increase the reaction. And then if temperature increases and the other variables are constant, then that will increase the rate at which the reaction happens.”

We summarize the major findings:

- Most students used linear causal mechanisms to explain the phenomenon even as they constructed dynamic time-based models.
- Two important factors—students’ iterative model evaluation and teacher prompts—resulted in the development of students’ ability to represent dynamic features in their model, which led to thinking of the phenomenon in terms of change over time, though to a limited extent.
- When students noticed the difference between real-world data and the model output, the model revision that followed focused on a single relationship and not on the interconnections between the system’s components.
- Students’ epistemological assumptions about computational modeling may have dictated the way they considered and interpreted real-world data.

## Discussion

In this study, we focus on the opportunities and challenges students encounter while constructing system dynamic models to make sense of a phenomenon. We observed challenges in modeling practices, reasoning regarding change over time, and using real-world data to revise models.

Based on other studies that describe the challenges individuals experience when required to reason in terms of change over time (Cronin et al., 2009; Sweeney & Sterman, 2000), we were interested in knowing to what extent a system dynamics modeling approach can support students and what challenges remain. Similar to the findings reported by Sander et al. (2006), we noticed that students start constructing their models in a linear causal chain fashion. This demonstrated students’ tendency to identify different causal relationships in which components do not accumulate or deplete over time. Such a model would not demonstrate change over time. Moreover, we identified that setting “time” as a distinct variable might lead to fallacious reasoning, in which time is the cause for the chemical reaction. That provides another example of how linear causal thinking can lead to erroneous reasoning and conclusions (Assaraf & Orion, 2005; Chi, 2005; Grotzer et al., 2013).

However, despite students’ inclination to generate a linear causal chain rather than a dynamic representation of a chemical reaction, the affordances of the modeling environment eventually allowed most students to adopt a time-based modeling approach. Moreover, we show that a shift to a time-based representation leads to a change in the terminology students use to explain the phenomenon. Therefore, we see our contribution in presenting a case in which system dynamics modeling, informed by the modeling process, can potentially shift the way students think about a phenomenon from linear causal reasoning to time-based reasoning. Furthermore, we show that the iterative nature of the modeling process and the opportunities students received to evaluate and revise their models throughout the unit supported that shift.

It is essential to emphasize that the simulation feature, which allowed students to test and evaluate their model, does not come naturally to students, as students are inclined to rely more on the structure of the model rather than on its behavior (indicated by the simulation). We observed that teachers’ support in encouraging their students to simulate their model further sustained students’ self-initiated simulations. This observation echoes similar findings that show that teacher support is essential to promote the evaluation and revision of models (Komis et al., 2007) and, furthermore, points out the importance of supporting students in developing agency in evaluating and revising models as crucial in constructing usable models (Reeve & Tseng, 2011).

One of the significant advantages of computational models is the ability to compare the model behavior to real-world data, which allows one to revise their model in the case of incongruence. Unfortunately, this is not always the case, as some students do not understand the underlying nature of modeling and the use of data. We show a case in which this comparison is made superficially, with no reference to the mechanism that underlies the phenomenon. Similar to Sins et al. (2009), we show that students tend to focus on fitting the model to real-world data rather than using the model to comprehend the phenomenon. In addition, we show that students tend to apply direct causal relationships as a means to fit the model to the data rather than investigating the interconnections of the system as a whole. In that sense, the affordances of system dynamic modeling are limited in scope unless students experience more scaffolds that support them in shifting from linear causal thinking to systems thinking that examines the interconnections between variables that lead to emergent behavior.

Students’ tendency to apply linear causal thinking across modeling practices should not come as a surprise, as it is unrealistic to think that a short intervention would profoundly affect habits of mind that have been encouraged since kindergarten, if not earlier. Indeed, other scholars have pointed out that K-12 science is focused on linear causal thinking, which results in difficulty explaining dynamic, complex phenomena (Plate, 2010; Raia, 2005). Although linear causal thinking is a valuable strategy to start making sense of a phenomenon, it is not sufficient for understanding complex systems and their behavior over time. However, we suggest that even the slight change students demonstrated in reasoning in terms of change over time while constructing system dynamics models is a promising avenue that can lead to desired outcomes.

In addition to linear causal thinking that may hinder the consideration of interconnectivity between system variables, students’ understanding of how to construct and interpret graphs can account for the challenges students face in comparing their model output (represented in a graph) to real-world data. Researchers report on challenges students face when interpreting graphs (Chinn & Brewer, 1993; Glazer, 2011). Comparing graph trends, in particular, is considered an advanced graph interpretation competency (National Council of Teachers of Mathematics, 2000; Wainer, 1992). It might be the case that the students in our study did not have many opportunities before this unit to engage in such a high level of graph interpretation and, therefore, lacked the ability required to manage this task.

The interviews revealed different underlying epistemologies that drove students’ revision process to reconcile the discrepancies between the model behavior and the experimental data. We noticed two different epistemologies regarding the modeling practice of evaluating and revising. The first epistemology, elicited in two of the four participant groups, assumed that the model needs to match real-world data (assuming it is trustworthy), rendering a necessary revision of the model. This notion implies that those students perceive the model as an abstraction of reality. Although this epistemological stance drove the students to revise their model to match experimental data, it was done in a superficial manner, with no consideration of the mechanism that underlies the behavior of the system.

We show that students’ describing their model as a “good enough match” when they compared it to real-world data might be due to the fact that students perceive the model as an absolute truth. Similar to findings reported by Cheng and Lin (2015), these results highlight the importance of the teacher and the curriculum providing synergistic support (Tabak, 2004) that specifically address the nature and purpose of models and the use of real-world experimental datasets in validating the usability of the model.

Unfortunately, the teachers did not provide students with sufficient support in using real-world data. The teachers encouraged the students to compare the data to the model and helped students to recognize the model output that they needed to compare with the real-world data; however, they did not provide the students with sufficient prompts that encouraged them to ponder the mechanism that would result in a matching system behavior and take a systems thinking approach that considers the interconnectivity between variables.

Therefore, like other scholars, we emphasize the need to develop strategies that teachers can apply to support students in using real-world data in a modeling context and explicitly address the epistemological assumptions that underlie students’ evaluation and revision of models (Komis et al., 2007; Schwarz et al., 2012). For instance, teachers could present to students previously built models of everyday phenomena that do not behave as expected and ask students to figure out what is not working in the model (e.g., the amount of money in the bank and what affects it over time) and revise the model in a way that meets expectations.

We argue that students’ challenges using real-world data partly hold students back from applying a feedback mechanism to explain the phenomenon. There is evidence in the literature for the challenges students face in understanding and using feedback mechanisms to explain phenomena (Tripto et al., 2013). Our curriculum, aligned with that challenge, was designed in such a manner that experimental data would encourage students to think about an alternative mechanism that would align with the expected behavior of the system. However, students’ revision of the model to fit real-world data, without accounting for the mechanism that underlies the behavior of a system, did not bring about a reasoning that goes beyond linear causal thinking. Hence, student models ultimately lacked feedback mechanisms.

## Research Limitations

We acknowledge several limitations in this research. First, the student population was not fully representative of the USA as a whole; the students who participated in our study were enrolled in a charter school based on their above-average performance in STEM subjects. Second, the number of students observed was insufficient to draw general conclusions. Third, SageModeler as any tool has its limitation. We acknowledge that in the context of chemical kinetics, the tool is limited in supporting students in fully understanding mechanism at the microscopic level, so we used simulations that demonstrate the behavior of particles at the microscopic level. Additional research needs to be done to understand if and how using simulations complements students’ modeling practices, and in particular how to support students in moving across the microscopic and macroscopic levels while applying ST. Finally, there may be aspects of the enacted unit that pertain specifically to the study of chemical kinetics and to modeling the rate of chemical reactions that pose unique challenges for students, including those that might be non-existent or negligible in other contexts.

## Conclusions

This study highlights some of the opportunities and challenges students encounter when building system dynamic models as they engage in modeling practices. This study contributes to the call for supporting students in dynamic thinking and mechanistic reasoning in the context of complex systems (Krist et al., 2019). We describe some success in students’ reasoning regarding change over time across modeling practices. We present the potential of using a system dynamics modeling approach to support students in thinking about change over time, which has been documented as a challenging ST aspect for students (Grotzer et al., 2013). We also show that students can represent some scientific principles in a dynamic model, making the case for the system dynamic approach as a viable way to support students in making sense of a phenomenon.

We also broaden the scope of challenges students face while engaging in system modeling to make sense of complex phenomena, including those that exhibit exponential, logarithmic, oscillating, or other non-linear behaviors. Our findings show that students tend to use linear causal relationships based on their prior academic experiences (Berkant, 2009; Cronin et al., 2009; Sweeney & Sterman, 2000) rather than using other mechanisms such as feedback. If we wish to involve students in making sense of complex phenomena and to prepare future citizens with the means to approach complex systems to find solutions to problems, we need to provide them with opportunities to move beyond linear causal thinking and adopt thinking in terms of change over time. Furthermore, feedback should be explicitly addressed as an underlying mechanism that explains systems behavior in everyday life to deepen and advance dynamic time-based reasoning. Most students’ default approach in conceptualizing systems focuses on linear causal chains, which as we showed, can present an obstacle when designing models of dynamic systems.

To summarize, our research suggests an avenue that supports students in the ST aspect of thinking in terms of change over time. We also present evidence that evaluating and revising a model using real-world data is not intuitive for high school students, even in a relatively supportive teaching and learning environment. In addition, we show that supporting student interpretation of data and graphs requires an attendance to underlying epistemological assumptions students have about using computational models. The findings in this study provide a foundation for further research on how teachers can support students in making sense of challenging ST aspects essential to making sense of dynamic systems, using computational system models aligned with the modeling process and its practices. An improved understanding of how to harness the use of modeling practices to support students in aspects of ST can lead to the development of new curriculum materials, assessment strategies, and scaffolds teachers can use to improve student engagement, understanding, and problem-solving skills.

## Figures and Tables

**Fig. 6:**
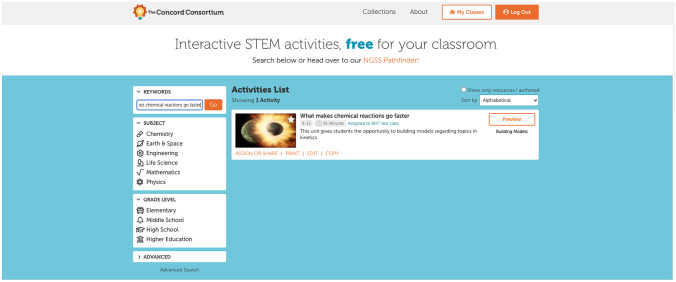
Access page on the concords consortium STEM research finder

**Fig. 7:**
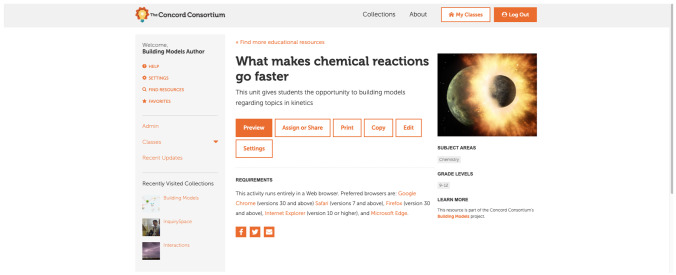
A webpage that directs the user to the unit

**Fig. 8:**
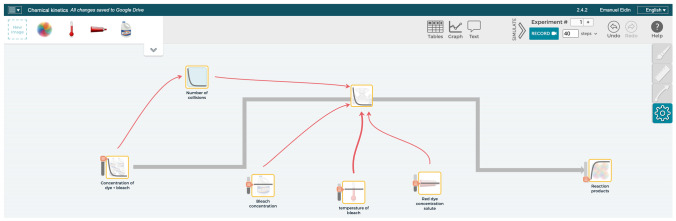
An example for a model that represents the phenomenon. One can notice the exponential decrease in the rate of reaction represented as a valve

**Fig. 9:**
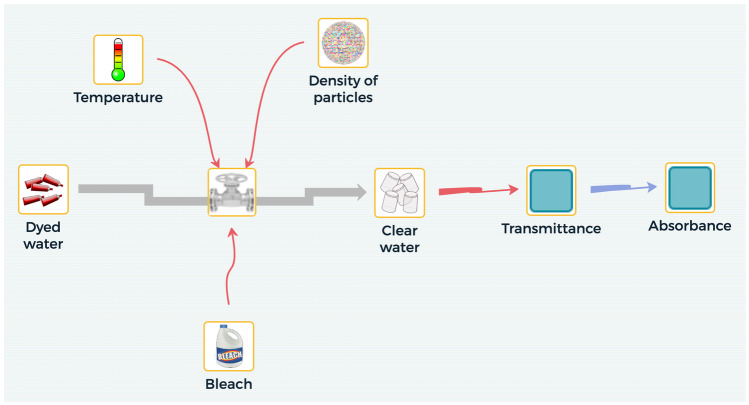
The last model revision of a pair of students

## Appendix 1. Students’ Interactive Unit

To access the interactive environment students have experienced, go to learn.concord.org.

Enter in search by keyword box- “what makes chemical reactions go faster” and click “enter” (Fig. 6).

The next page should appear. Click the unit’s headline.

After you clicked the headline, the next page should appear; click the preview box, and you enter the interactive environment the students have experienced.

## Representation of the Phenomenon in SageModeler

For a better understanding of the educational context and how one can represent change over time of a chemical reaction, we briefly explain how the reaction between bleach and dye is represented in SageModeler. To see behavioral change over time, one must utilize a unique variable known as a “collector.” Like a stock in a “stock and flow” model, a collector accumulates changes associated with successive calculations; as such, changes over time become evident in the model behavior. When performing a simulation, each variable node displays a graph that represents change over time. For example, a flat line indicates that a variable exhibits no change over time (e.g., temperature’s solution in Fig. 8). To represent change over time, one needs to set at least one variable as a collector (e.g., reaction products in Fig. 7). As mentioned previously, prior to beginning the kinetics unit, students completed an introductory unit on building dynamic models in SageModeler and interpreting model output. The teacher explicitly mentioned that the model’s output represents change over time.

## Appendix 2. More information About SageModeler

For more information and a first-hand experience with SageModeler, go to https://sagemodeler.concord.org/.

## Appendix 3. Scoring Rubric of Students’ Models

For the “defining system boundaries” practice, models were scored according to four criteria: (1) the number of *key variables*, (2) the number of *content-inappropriate variables*, (3) the number of *model-inappropriate variables*, and (4) the number of *irrelevant variables*. Each of the criteria was scored separately and then combined into a composite score (Table 5).

- *Key variables*: Quantifiable model components that are essential to make sense of the phenomenon and without which the system mechanism cannot be explained (Bielik et al., 2020).
- *Content-inappropriate variables*: Variables that do not belong to the system under study or whose inclusion results in the loss of coherent meaning.
- *Model-inappropriate variables*: Variables named such that they represent other entities (e.g., an event or object). Variables that are incongruent with a time-based model are also included under this category.
- *Irrelevant variables*: Variables that are not necessary to explain the phenomena yet can still be found within the system boundaries.

**Table 5:** Define the boundaries of the system-scoring variables in model

**Code**	**Definition**	**Identification**	**Score**	**Example from kinetics unit**
Key variable (KV)	Variables that are crucial for the investigated system	Predetermined for each model based on curriculum	+1	Reactants, products, bleach, temperature, absorbance/transmittance, number of collisions
Irrelevant variables (IRV)	Variables that are not key to the system but are not a mistake	Predetermined for each model based on examples that are agreed between raters	0	pH, dye, particle size? * If both absorbance and transmittance are included, one of them is irrelevant
Inappropriate variable in Content-level (IAV-C)	Variables that are wrong in the content-conceptual level	Not predetermined	−1	
Inappropriate variable in Model-level (IAV-M)	Variables that are wrong in the model-conceptual level	Not predetermined	−1	Time, multiple colors of dye variables

Defining a variable as collector does not affect this category

To assess the modeling practice of “setting relationships,” we evaluated each relationship that appeared in the model according to the type of relationships that are possible in the software. As noted, SageModeler facilitates the setting of semi-quantitative relationships between variables, including several options for setting the magnitude of the impact of each of the variables on one another. For example, if students decided that an increase in one variable had an impact on another variable, they could set the magnitude that defines this relationship as (1) about the same (a linear graph), (2) a little (a linear graph with a gentle slope), (3) a lot (a linear graph with a steeper slope that levels off), (4) more and more (an exponential graph), or (5) less and less (a logarithmic graph). Each of these categories represents the type and quality of a given relationship. Inappropriate relationships were given a negative score, while appropriate relationships received a positive score. See Table 6.

**Table 6:** Design and construct the model structure- scoring relationships between variables

**Relationship and score**	**Variable to variable or variable to valve**	**Collector to collector**	**Variable to collector**
Right relationship, right type (+1)	-	-	Right relationship
Right relationship, wrong type (+0.5)	Wrong by the same/little…	Wrong by add/subtract/transform	-
Wrong relationship (−1)	Wrong increase/ decrease	Two collectors are not of the same entity	Wrong by add/subtract
Reverse direction (−1)	Reverse arrow	Relationship between collectors should be the opposite	-
Number of connections between irrelevant variables (0)	-	-	-

For the “use of the model,” we evaluated each model for the representation of key scientific ideas used to explain the phenomenon based on model structure and output. We sorted the results into four categories, including (1) models that accurately explained and/or predicted the phenomenon and included correct trends and magnitudes, (2) models that partially explained or predicted the behavior of the phenomenon (i.e., correct trend but incorrect magnitude), (3) models that included key structural components but did not explain or predict the phenomenon (i.e., an incorrect trend), and (4) models that did not include key structural components and as such neither explained nor predicted the phenomenon (i.e., no trend predicted) (Table 7).

**Table 7:** Using the model to explain or predict

**General description**	**Indicator in the kinetics unit**	**Score**
Model explains/predicts accurately the phenomena. Includes right trend and magnitude	Model predicts exponential behavior of decrease in reactants/ absorbance over time (Right trend, right magnitude)	4
Model partially explains/ predicts accurately the phenomena (right trend, wrong magnitude)	Model predicts a linear drop in reactants/ absorbance	3
Model includes key structural components but does not explain/ predict the phenomena	Model addresses change over time of reactants and products, but the prediction shows an opposite trend line	2
Model does not include key structural components and therefore does not explain/ predict the phenomena	Model does not predict change over time of reactants and products, a static model in a dynamic system	1

Attached is our scoring rubric for the students’ models.

### Description of the Evaluation Process of Students’ Models

To better understand the evaluation process of the models and the modeling practices, we will use the next model example in Fig. 9 of a student to demonstrate the process.

#### Evaluation of “Defining System Boundaries”

To evaluate the “defining system boundaries” practice, we first count the number of key variables. In this case, most key variables are addressed. Students included the reactants, the products, temperature, concentration, and the empirical representation of reaction rate (absorbance). We acknowledge that labeling the variables could have been more accurate; however, we decided not to score the wording of variables as long as the students’ intent was clear. For example, it would have been more accurate to label the variable “amount of bleach” rather than simply “bleach”; yet it is clear from the model structure and relationships that the variable refers to the amount or concentration. We believe that addressing errors in wording and labeling misses the purpose of evaluation and shifts the focus away from evaluating students’ ST as manifested in the modeling practice being scored.

In this model, we find one irrelevant variable. Since both transmittance and absorbance are expressions of Beer-Lambert’s Law, it is sufficient to address either of them; including both is unnecessary. (The spectrophotometer used in the experiment showed results of transmittance and absorbance.)

The model includes one inappropriate variable, “density of particles.” Because density does not contribute to the explanation of the driving question, it is inappropriate. Students received scores for including key variables and were penalized for an inappropriate variable. Students were not penalized for having irrelevant variables. Therefore, the score for including five key variables is 5, but one point was deducted for including an inappropriate variable. The composite score is 4.

#### Evaluation of “Setting Relationships”

Links between standard variables and flow control variables (represented by the valve symbol) are addressed as “variable to variable” relationships. The valve references a water metaphor, in which a flow or rate of transfer from an entity in one form to the same entity in another form is controlled by the valve (Forrester, 1994; Sweeney & Sterman, 2000). In this model, there is a flow from reactants (i.e., “dyed water”) to products (i.e., “clear water”). Students constructed three relationships that point to the valve. We describe how we evaluated each relationship.

- The relationship between “bleach” and the valve is correct. An increase in reactants’ concentration causes the rate of reaction to increase proportionally.
- The relationship between “temperature” and the valve. The relationship has the appropriate direction of causality, but it is not specified correctly. An increase in temperature will cause the rate of reaction to increase exponentially. However, given that the students were not provided with any evidence during the unit that demonstrates the relationship between temperature and rate of reaction, aside from the general trend, we gave the maximum score if students could point to the right direction of causality.
- The relationship between “density of particles” and the valve. Because the “density” variable is inappropriate given the context and purpose of the model, the relationship itself is irrelevant, and we do not include that relationship in our scoring.

The causal chain relationship between “clear water” (which represents products) and “transmittance” and between “transmittance” and “absorbance.” Because transmittance is an irrelevant variable, we omitted the relationship from the causal chain and evaluated the relationship between “clear water” and “absorbance.” This relationship has the right causal relationship but the wrong type of relationship. The amount of “clear water” (products) will reduce the absorbance, though not in “more and more” type of relationship; the correct relationship is “about the same.”

Each relationship that was partially correct received 0.5 points (out of a possible 1 point). Because the “density of the particles” variable was already penalized for being inappropriate, we avoided double penalization and did not score that relationship.

Evaluating the “collector-to-collector” relationship. To set this relationship, students need to think of the reactants as a collection of particles that turn into a collection of product particles and set the relationship between the two that will cause that change from one to the other over time. In this case, students created an appropriate transfer link between variables that represent the reactants and products, for which they received 1 point.

#### Evaluation of “Use of the Model”

This model partially explains or predicts the behavior of the phenomenon (i.e., correct trend but incorrect type of relationship). The model predicts that reactants and products will behave linearly. It does not include a feedback mechanism that would give rise to an exponential decay of reactants as we would expect.

Using the rubric to evaluate student models provided us with a means to track learning quantitatively as students progressed through the different modeling practices and across different model iterations. Our goal was to indirectly measure student engagement in ST and CT through the scoring of modeling practices, given our previous proposition that each practice necessarily involves specific aspects of ST and CT.

## Appendix 4. Interview Protocol

Introduction:

Script:

This will be said to the class as a whole

Hi *X (state the student's name), my name is X (state your name) and I’m a researcher at Concord Consortium and I work with the Michigan State University team. I’ll be interviewing several of you to learn about your experiences with the “–-” unit. I’m interested in your experiences of learning the “–-” unit, and I would like to ask you a few questions about it, if that’s ok (wait for their consent).*

This will be said in interview

*Hi (state the student’s name). Thank you so much for agreeing to be interviewed. Your feedback will help us improve the software and make it better. Please be honest and open in your responses, they will only be used for our evaluation of the modeling tool and will not affect your grades in any way.*

### (Follow the Script, Try to Remain Neutral, Don’t Give Feedback About Correctness)

Neutral probes

- What are you thinking? Can you think aloud?
- Can you say more about that?
- What do you mean?
- Why do you think that?

## Part 1- Planning (Problem Decomposition/System Thinking)

### Before Navigating to the Student Pages

1. Can you tell me what the unit was about? What question were you trying to answer?
2. How do you think the model that you built could help someone to answer the question you are trying to answer? (combined w the next question below)

## Part 2- Modeling Practice and Model-based Explanation Questions

### Ask the Student to Recall Developing and Revising Their Models Using SageModeler.

With their final SAGE model visible

1. Can you tell me the story of your model? How does it answer the driving question? Can you walk me through your model?
  1. This one (collector) has little boxes in it and this one (variable) doesn’t. Why did you choose to make this one a collector (with little boxes)? [For Model 1 only, which is all collectors, skip this question] How (or Why) did you define collectors among your variables (ideas)?
  2. Which variables and relationships are most responsible for changes in the collector or collectors? (*Point to a collector that has something pointing to it)* What is causing this to change?
  3. [If they do not refer to the flows when answering *b*] What do you think these pipes mean? (*point so that they know what I mean)*
  4. (*open box w description of link w variable directly pointing into collector where units don’t match*) What does this sentence mean?
  5. [If the model has red arrows or blue arrows] What do you think these arrows mean?
  6. (open box w description of one of the arrows, read sentence) Why did you choose this one? (open drop-down menu to show the other choices)
  7. Can you tell me what transmittance means to you? What about absorbance?
2. [Different for each model]

## Model 1

- Do you think there need to be any other arrows pointing to this pipe?
  - What do you mean that amt of dye transfers to absorbance?
  - When transmission goes up, does absorbance go up?

## Model 2

- Really interesting that everything is pointing to this valve. Could have pointed to the collectors, for example. Can you explain your choice to do it this way?
- (Graph: hide one run, then the other, and ask difference between them)
- When absorbance goes up, does transmittance goes up?

## Model 3

[Two things pointing to reaction rate. Do not have dye particles pointing in.]

- What things are influencing how fast the dye particles split? What about these orphan variables? Do they affect how fast? What happened when they were in your model?
  - Can you tell me what this graph shows about the phenomenon?

## Model 4

- Bleach affects rate, temperature affects rate. What about red dye? Does it affect rate?
- Would like to ask you several questions about absorbance and what influences it.
  - What do you think it means when two arrows point to the same thing? (absorbance) How does temp affect it? How does dye affect it?
  - Is there anything else that impacts absorbance? Directly or indirectly? In class, did you put in bleach and color goes away? How is this shown in your model?
  - How does temp affect absorbance? Long set of connections, short (indirect) connection, need both?

## All Models

- Did this model help you understand the phenomena? How?
- Your model indicates that an increase in temperature or concentration of bleach will increase the rate of the chemical reaction. Can you explain why these increase the rate? (edited)
- How does the absorbance/transmittance over time give an indication of the rate of a chemical reaction?

Let’s imagine for a moment. What if we change something in your model?

1. If we change the direction of this connection (*choose a connection that is not in a feedback loop)*, what does that mean? How does that change what happens?
2. If we remove this connection (*if there are feedback loops, choose one and point to a connection that would eliminate it*), how does that change the behavior of this collector? (*Point to a collector in the loop or chain.*)
3. If we add a connection here (*choose a place that would create a feedback loop*), what does your model predict would happen? Can you talk me through the effect of this change on your model?

## Part 3- Evaluation: Revise/Debug/Test

With their initial, revise, or/and final models visible.

1. How would you evaluate whether your model is good or not? Can you explain why you built your model in this way?
  1. *(Probe for this)*

With their revised parts in their models visible

2. Why did you revise (and please explain the revision) –– from the previous to the final models

### Part 4- Data Practices: Interpret/Analyze/Visualize

With their data table, experimental graph(s) and Sage model visible [no Sage graphs saved].

1. I see the graphs you generated from your experiments. Did you generate any from your model?
  1. If so, how did your model graphs compare with your experiment graphs? Can you tell me about that?

## Closing

Do you have any questions for me?

Thank you so much, this was very helpful.

END OF INTERVIEW FOR CHEM STUDENTS
